# Continuous Interscalene Brachial Plexus Blocks: An Anatomical Challenge between Scylla and Charybdis?

**DOI:** 10.3390/medicina60020233

**Published:** 2024-01-29

**Authors:** Rainer J. Litz, Georg C. Feigl, Daniel Radny, Thomas Weiß, Peter Schwarzkopf, Tim Mäcken

**Affiliations:** 1 Independent Researcher, 86199 Augsburg, Germany; rainer.j.litz@gmail.com; 2 Institute of Anatomy, University of Witten/Herdecke, 58455 Witten, Germany; georg.feigl@uni-wh.de; 3 Department of Anaesthesiology and Intensive Care Medicine, St. Josef-Hospital, Ruhr-University Bochum, 44791 Bochum, Germany; daniel.radny@ruhr-uni-bochum.de; 4 Department of Anesthesia and Intensive Care Medicine, Thurgau Cantonal Hospital, 8596 Münsterlingen, Switzerland; thomas.weiss@stgag.ch; 5 Clinic for Anesthesiology, Intensive Care, Palliative and Pain Medicine, Sana Hospital Leipziger Land, 04552 Borna, Germany; peter.schwarzkopf@sana.de; 6 Department of Anaesthesiology, Intensive Care and Pain Medicine, BG University Hospital Bergmannsheil, 44789 Bochum, Germany

**Keywords:** interscalene brachial plexus block, interscalene catheter, ultrasound, scalenovertebral triangle, shoulder surgery, catheter malposition

## Abstract

Brachial plexus blocks at the interscalene level are frequently chosen by physicians and recommended by textbooks for providing regional anesthesia and analgesia to patients scheduled for shoulder surgery. Published data concerning interscalene single-injection or continuous brachial plexus blocks report good analgesic effects. The principle of interscalene catheters is to extend analgesia beyond the duration of the local anesthetic’s effect through continuous infusion, as opposed to a single injection. However, in addition to the recognized beneficial effects of interscalene blocks, whether administered as a single injection or through a catheter, there have been reports of consequences ranging from minor side effects to severe, life-threatening complications. Both can be simply explained by direct mispuncture, as well as undesired local anesthetic spread or misplaced catheters. In particular, catheters pose a high risk when advanced or placed uncontrollably, a fact confirmed by reports of fatal outcomes. Secondary catheter dislocations explain side effects or loss of effectiveness that may occur hours or days after the initial correct function has been observed. From an anatomical and physiological perspective, this appears logical: the catheter tip must be placed near the plexus in an anatomically tight and confined space. Thus, the catheter’s position may be altered with the movement of the neck or shoulder, e.g., during physiotherapy. The safe use of interscalene catheters is therefore a balance between high analgesia quality and the control of side effects and complications, much like the passage between Scylla and Charybdis. We are convinced that the anatomical basis crucial for the brachial plexus block procedure at the interscalene level is not sufficiently depicted in the common regional anesthesia literature or textbooks. We would like to provide a comprehensive anatomical survey of the lateral neck, with special attention paid to the safe placement of interscalene catheters.

## 1. Introduction

**Figure 1:**
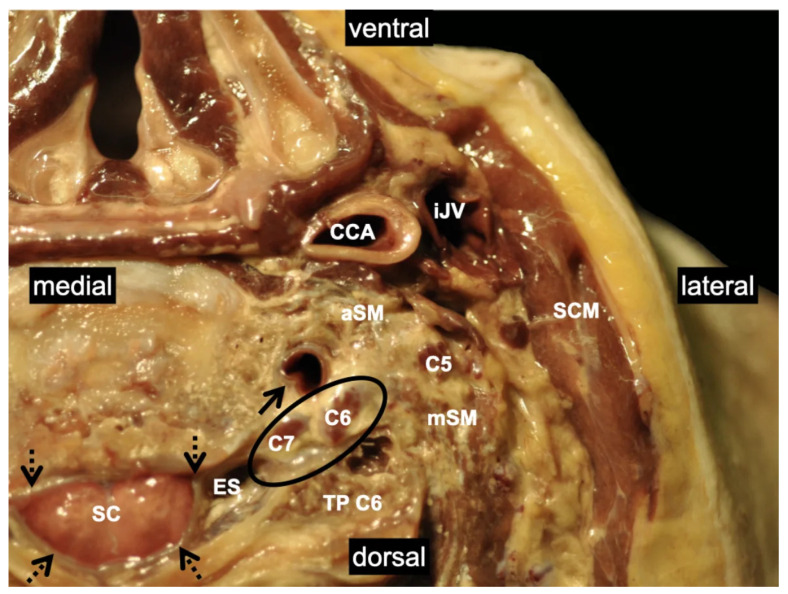
Transverse section of the lateral neck at the level of the larynx illustrating the close relationship between the spinal canal and the ventral rami of C5, C6, and C7. Catheter advancement along the nerve course toward the neuroforamen may explain intrathecal misplacements. The black circle indicates the close relationship of the ventral rami to the epidural space and the spinal cord. Dotted black arrows mark the dura mater. Non-dotted black arrow: vertebral artery; C5: ventral ramus of the 5th spinal nerve; C6: ventral ramus of the 6th spinal nerve; C7: ventral ramus of the 7th spinal nerve; CCA: common carotid artery; ES: epidural space; aSM: anterior scalene muscle; mSM: middle scalene muscle; SCM: sternocleidomastoid muscle; SC: spinal cord; TP C6: transverse process of the 6th cervical vertebra; iJV: internal jugular vein. The details of the body donor’s embalmment, preparation, and dissection are provided in Appendix A.

The use of interscalene brachial plexus blocks has been an established procedure for decades to provide intra- and postoperative analgesia for patients undergoing shoulder surgery. Although often regarded as a simple procedure [1], this technique is associated with specific side effects and complications. The underlying reasons are certainly not known to all users [2,3]. Typical undesired side effects such as phrenic nerve palsy, Horner’s syndrome, or recurrent nerve palsy are commonly observed [3]. Among them, the frequently observed ipsilateral phrenic nerve block and hemidiaphragmatic paralysis (HDP) are considered the most undesirable side effects. The use of ultrasound allows clinicians to reduce local anesthetic (LA) volumes [4,5,6], concentrations [7,8], and/or LA injections [9] outside the interscalene gap. All these strategies can reduce the incidence of HDP but not completely negate it. Consequently, numerous alternative techniques, such as C7 nerve root [10], suprascapular [11], upper-trunk [12], and costoclavicular brachial plexus block [13], have been described and extensively discussed [14,15]. They all provide acceptable postoperative analgesia but not to the same degree as interscalene blocks (ISBs), which are conceptional from an anatomical view. Furthermore, they cannot reduce the risk of HDP to zero, which is in demand for patients with severe pre-existing pulmonary disease. The side effects of a single-injection interscalene block (ISB) can be explained by uncontrolled LA spread, especially with larger LA volumes, high injection pressure, or a medially directed canula placement toward the neuroforamina. Besides relatively mild side effects, serious complications such as intrathecal [16] or intravascular injections [17] or damage to the spinal cord [18] have been reported in relation to single-injection ISB. Catheter techniques for continuous postoperative analgesia are even more challenging. Despite uneventful initial LA injection before catheter placement, life-threatening and fatal complications have been reported following intrathecal [2,19,20], epidural [21], or intravascular [22,23] catheter positioning. Symptom onset for misplaced catheters can occur immediately after injection but also with a delay in non-monitored patients [24]. The underlying mechanisms can be explained by extensive catheter threading beyond the correctly placed canula [2] or by late dislocation [25,26]. Because of the good analgesic effects of interscalene brachial plexus blocks, whether administered as a single injection or continuously, they remain the standard of care for many anesthesiologists. To understand the mechanisms of potentially severe side effects, profound anatomical knowledge of the posterior triangle and the adjacent space of the neck is mandatory. For example, the epineurium of the ventral rami of the spinal nerves is the continuation of the dura mater. One could argue that the interscalene brachial plexus block may be considered a central neuraxial rather than a peripheral nerve block (Figure 1). The reports of life-threatening cases of neuraxially misplaced interscalene catheters confirm these propositions. We would like to discuss, from an anatomical basis, why the safe use of interscalene catheters is a balance between high analgesia quality and complications, much like the passage between Scylla and Charybdis.

## 2. Relevant Anatomy for Interscalene Brachial Plexus Blocks

### 2.1. Innervation and Formation

The brachial plexus originates from spinal segments C5 to Th1 and innervates the upper extremity and parts of the trunk wall. The plexus runs through four defined anatomical regions according to its course from proximal to distal directions: (1) the scalene hiatus (the hiatus scalenorum posterius), (2) the posterior or lateral neck triangle (trigonum colli posterius sive laterale, the posterior cervical triangle), (3) the infraclavicular region (trigonum deltoideopectorale sive, Mohrenheim’s fossa or groove), and (4) the axillary fossa.

**Figure 2:**
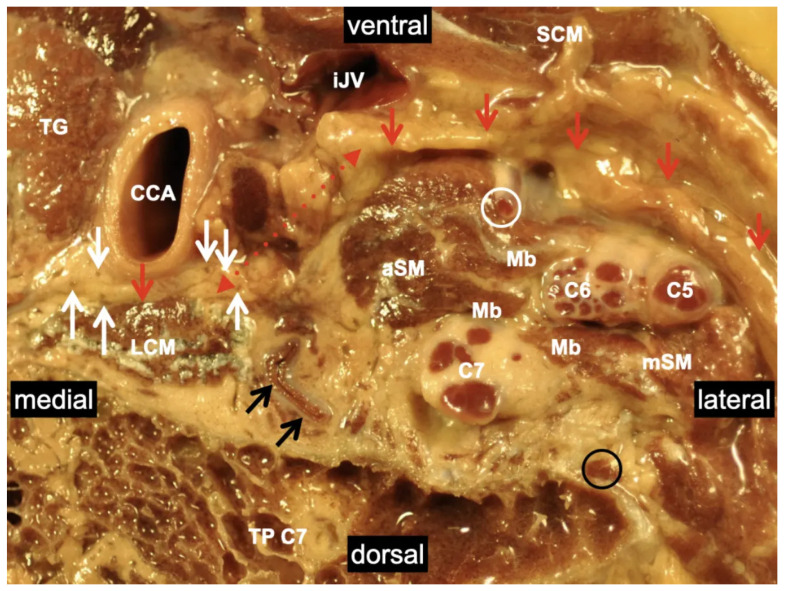
Transverse section of the lateral neck region showing the anatomical relationships within the compartment of the brachial plexus. A muscular connection separates the ventral rami from C5, C6, and C7. The phrenic nerve is depicted deep to the prevertebral fascia within the same compartment as the brachial plexus. The gap is narrowed by fat and connective tissue. Red arrows mark the prevertebral fascia; black arrows mark the vertebral artery; white arrows mark the sympathetic trunk; white circle: phrenic nerve; black circle: dorsal ramus of the 6th spinal nerve; C5: ventral ramus of the 5th spinal nerve; C6: ventral ramus of the 6th spinal nerve; C7: ventral ramus of the 7th spinal nerve; CCA: common carotid artery; Mb: muscle bridges; LCM: longus colli muscle; aSM: anterior scalene muscle; mSM: middle scalene muscle; SCM: sternocleidomastoid muscle; TP C7: transverse process of the 7th cervical vertebra; TG: thyroid gland; iJV: internal jugular vein. Note: the continuity of the prevertebral fascia of the body donor was accidentally interrupted via dissection (dotted red arrows). This location represents the connection to the Danger Space [27]. Details of the body donor’s embalmment, preparation, and dissection are provided in Appendix A.

The fila radicularia arise from the spinal cord and form the radix anterior and radix posterior, which then form the actual spinal nerves in the spinal canal. In the cervical region, the spinal nerves emerge, after their formation, from the spinal canal and on through the neuroforamina (intervertebral foramina). Immediately after their ascent from the neuroforamen, they split into a larger ventral ramus and a smaller dorsal ramus (Figure 2). The brachial plexus is formed in the posterior scalene gap by the ventral rami of the spinal nerves. The dorsal rami supply the neck muscles with motor activity and the skin of the neck and occiput with sensory activity. They are not involved in the formation of the brachial plexus. The distance to the plexus formation is often less than 1 cm after the splitting of the spinal nerves.

In the scalene gap, the brachial plexus is located deep to the prevertebral fascia between the anterior and middle scalene muscles in a compartment that is rich in nerves and vessels. When observed in ultrasound images, the ventral rami are often described as roots; however, the anatomical term roots describes the anterior and posterior radices occurring before the formation of the spinal nerves. They are located more centrally and are not visible through ultrasound as they are covered by the vertebral arch of the bony spinal column. After they exit the neuroforamina, the ventral rami can be visualized sonographically.

### 2.2. Anatomical Variations in the Scalene Gap

**Figure 3:**
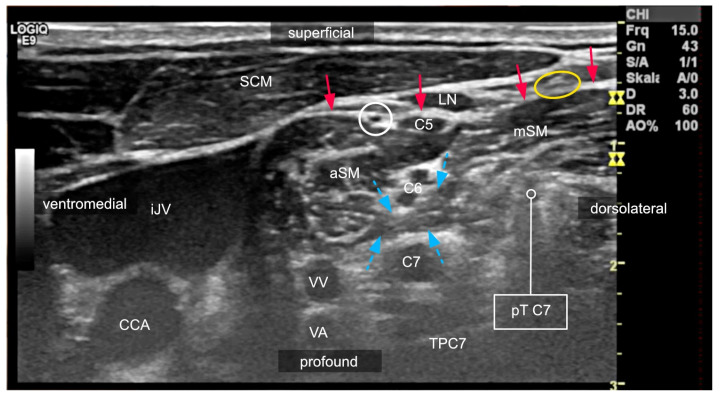
Sonogram of the lateral neck region at the level of the 7th cervical vertebra captured to demonstrate problems of LA distribution. Nerves of the brachial plexus are deep to the prevertebral fascia (red arrows), and the supraclavicular nerves from the cervical plexus (not depicted) are superficial to it. Muscle bridges between anterior (aSM) and medius scalene muscle (mSM) act as mechanical barriers to LA spread (blue arrows). In this patient, local anesthetic would likely cause phrenic nerve (white circle) palsy if injected close to the ventral ramus of C5 due to its direct proximity to the phrenic nerve within the same compartment. The yellow circle marks a supraclavicular nerve. The vertebral vessels (VA: vertebral artery; VV: vertebral vein) are in close proximity to the 7th spinal nerve (C7). They and the spinal nerve at the level of the neuroforamen will be subject to injuries if a block with a needle trajectory planned according to Winnie is carried out. C6: ventral ramus of the 6th spinal nerve; SCM: sternocleidomastoid muscle; TPC7: transverse process of the 7th cervical vertebra; pTC7: posterior tubercle of the 7th transverse process; LN: lymph node; CCA: common carotid artery; iJV: internal jugular vein.

As a consequence of embryonic development and the principle of segmental innervation, fibers of C4 (cranial fixation) and T2 (caudal fixation) may be involved in plexus formation as well. In addition to variants of plexus formation, variants of the scalene musculature [28,29,30,31,32] and/or vessels [33] can frequently be found. There are often muscular connections between the anterior and middle scalene muscles. Another scalene muscle, the scalene minimus muscle, referred to in older nomenclature as the levator pleurae muscle [32], is frequently present. Such muscles may separate the plexus into different parts. In practice, such muscle formations, as well as the loose connective and fatty tissue located in the scalene gap (Figure 1), narrow the gap and make spatial LA distribution unpredictable or even prevent it from spreading around the plexus (Figure 3).

### 2.3. Variants of the Course of the Ventral Rami

**Figure 4:**
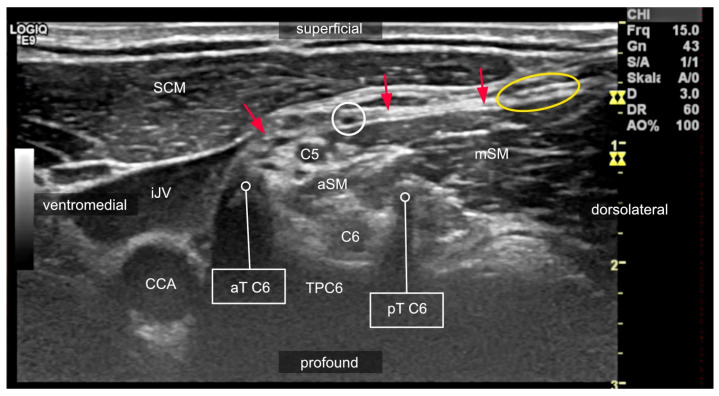
Sonogram of the lateral neck region at the level of the 6th cervical vertebra, demonstrating the variable course of the ventral rami (spinal nerves). In this patient, the ventral ramus of the 5th spinal nerve (C5) passes superficially to the anterior scalene muscle (aSM), while the ventral ramus of the 6th spinal nerve (C6) does not. Nerves of the brachial plexus were deep to the prevertebral fascia (red arrows), with the supraclavicular nerves from C4 running superficially to it (yellow circle). The phrenic nerve (white circle) is located deep to the prevertebral fascia on top of the fascia of the aSM. A phrenic-nerve-sparing brachial plexus block at this level is simply not possible. The ventral ramus of the 6th spinal nerve (C6) is depicted at its course out of the neuroforamen between the anterior tubercle (aT C6) and posterior tubercle (pT C6). Any needle advancement toward the neuroforamen carries the risk of nerve injury since the nerve at this position is not movable between the bony structures. SCM: sternocleidomastoid muscle; TPC6: transverse process of the 6th cervical vertebra; mSM: middle scalene muscle; iJV: internal jugular vein; CCA: common carotid artery.

In the scalene gap, the cranial plexus parts (C5 and C6) frequently disperse between the anterior and middle scalene muscles and often do not intersect the caudal parts of C7, C8, and Th1 (Figure 3). C5 may run anteriorly or through the anterior scalene muscle (Figure 3 and Figure 4), and C5 and C6 may also pierce the anterior scalene muscle together [32]. This explains the clinical observation of incomplete interscalene blocks regardless of whether neurostimulation or ultrasound guide techniques are used (Figure 3 and Figure 4).

### 2.4. Compartments of the Lateral Neck and Their Relevance to Side Effects

**Figure 5:**
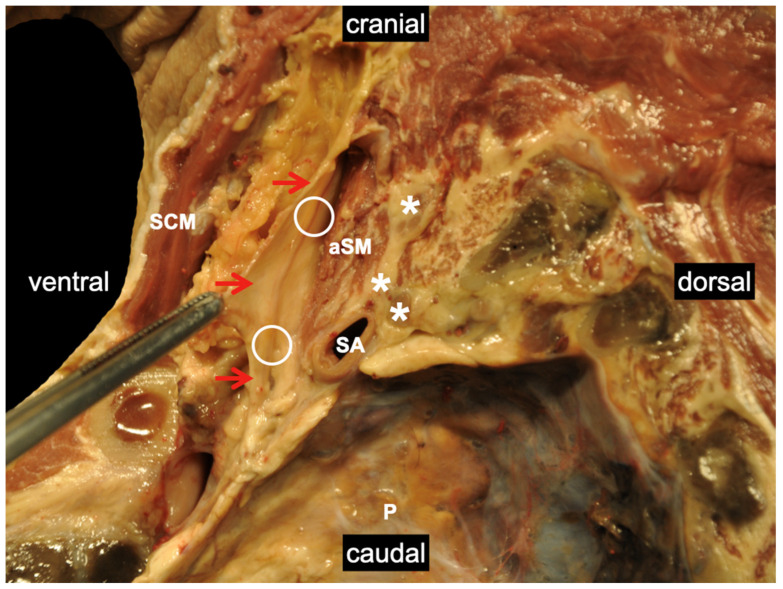
Sagittal section through the left lateral neck region (scalenovertebral triangle) including the pleural cavity. The phrenic nerve (white circles) is deep to the prevertebral fascia (red arrows). White stars mark the ventral rami of the spinal nerves, pointing out their close vicinity to the phrenic nerve, which explains why phrenic-nerve-sparing injection techniques are so difficult and why ventro-medial LA distributions should be avoided. SA: subclavian artery; P: pleura; aSM: anterior scalene muscle; SCM: sternocleidomastoid muscle. Details of the body donor’s embalmment, preparation, and dissection are provided in Appendix A.

In the interscalene gap, the brachial plexus lies deep to the prevertebral fascia, as do the ventral rami of C1 to C4 that form the cervical plexus. The phrenic nerve usually arises from C4 and occasionally, in part from C3 or C5 or even lower cervical segments. The nerve then runs covered by the prevertebral fascia on the ventral surface of the anterior scalene muscle. Figure 5 demonstrates the close vicinity of the brachial plexus to the phrenic nerve.

The sympathetic trunk is located medial to the scalene gap on the surface of the longus colli muscle. It is anteriorly attached and partially enveloped by the prevertebral fascia. If LA spreads medially deep or anterior to the anterior scalene muscle, it can disperse into the scalenovertebral triangle [31,34]. The triangle is bounded laterally by the anterior scalene muscle and medially by the longus colli muscle. The tip of the triangle is marked by the carotid tubercle (the anterior tubercle) of the sixth cervical vertebra. The base is defined by the subclavian artery or the cupula pleurae [35]. The large vessels, the common carotid artery, and the internal jugular vein, as well as the vagal nerve, are enveloped in this position by the carotid vagina and mark the ventral border.

Due to the presence of the pleural dome, the scalenovertebral triangle corresponds to a pyramidally shaped three-dimensional space. The prevertebral fascia surrounds the anterior scalene and the longus colli muscle. However, the prevertebral fascia does not separate these two muscles. Consequently, there is a connection from the scalenovertebral triangle to the dorsal prevertebral, the so-called Danger Space [27], which connects to the mediastinum.

### 2.5. Danger Space

The Danger Space constitutes a loose connective tissue space with a craniocaudal extension from the skull base to the diaphragm. This space contains the recurrent laryngeal nerve, parts of the sympathetic trunk and the vertebral vessels, autonomic fibers of the heart, and the phrenic nerve [27] (Figure 5). Therefore, LA spreading into this space can cause unwanted side effects like Horner’s syndrome or hoarseness.

### 2.6. Are Brachial Plexus Blocks Sufficient for Shoulder Surgery?

**Figure 6:**
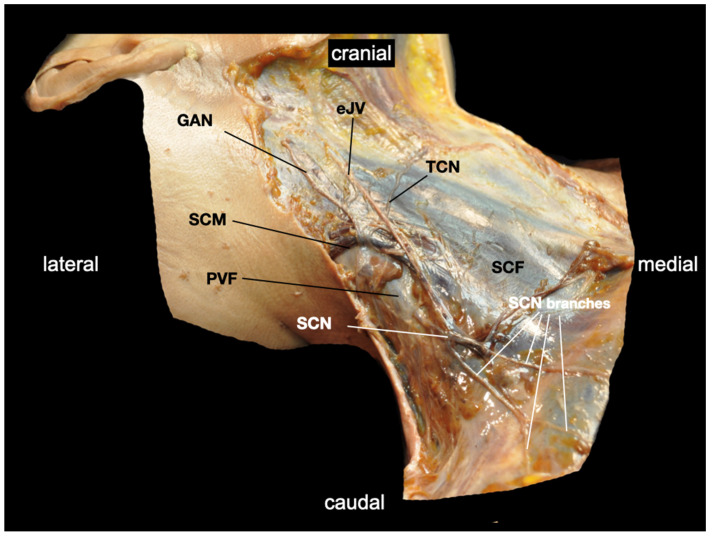
The dissection of the right lateral neck (ventrolateral view) demonstrates the peripheral course of the supraclavicular nerves after their exit through the prevertebral fascia (PVF). The PVF is depicted at the lateral border of the sternocleidomastoid muscle (SCM). This image clearly depicts the different compartments of the cervical and brachial plexus. Analgesia for skin incision or closure in shoulder surgery can only be achieved if the supraclavicular nerves are blocked superficial to the PVF, provided that the phrenic nerve can be spared. eJV: external jugular vein; GAN: greater auricular nerve; SCF: superficial cervical fascia; SCN: common trunk of the supraclavicular nerves; TCN: transverse nerve of the neck. Details of the body donor’s embalmment, preparation, and dissection are provided in Appendix A.

ISBs are usually applied for shoulder surgery, which ranks among the most painful surgical procedures [36]. The suprascapular nerve and the axillary nerve, both originating from the ventral rami from C5 and C6, which form the superior trunk, are the main suppliers of the shoulder joint. The lateral pectoral nerve, which originates from the lateral cord, is involved, and so too are the subscapular nerves [37,38]. Since the suprascapular nerve originates from the most proximal section of the brachial plexus (the superior trunk), the target for shoulder surgery must be the brachial plexus at the level of its trunks. This explains the superior analgesic effects of an interscalene or supraclavicular block. Furthermore, the cutaneous innervation of the shoulder cape is also mediated by the supraclavicular nerves originating from C4, like the phrenic nerve. They have a short course of up to 2 cm underneath the prevertebral fascia; they then penetrate the fascia and become superficial (Figure 6). After passing through the prevertebral fascia, they run in a different compartment, superficial to the brachial plexus. These anatomical facts indicate why they are unlikely to be blocked using peripheral techniques (such as a costoclavicular block). Therefore, if shoulder surgery is carried out solely with peripheral nerve blocks, brachial plexus blocks must be combined with a supraclavicular nerve block to anesthetize the skin for incision or closure.

## 3. Anatomical Challenge between Scylla and Charybdis

### 3.1. Local Anesthetics Volume

Clinically used LA volumes can spread medially to the anterior scalene muscle and reach vascular and nervous structures in the Danger Space and possibly the mediastinum. This spreading may include the recurrent laryngeal nerve, the cervical sympathetic trunk, nerves of the autonomic innervation of the heart, or the phrenic nerve [39]. Therefore, the anatomical conditions may explain the extensive side effects that are regularly reported in the literature with high LA injection volumes of up to 40 mL or even more [3]. Reducing the LA volume to 5–7 mL may reduce the incidence of side effects but cannot preclude them completely. A further reduction in the LA volume results in incomplete analgesia [40]. However, even with such small volumes, magnetic resonance imaging has demonstrated that the LA can reach the neuroforamina and the cervical epidural space [5]. These results were also shown in an anatomical study conducted on body donors [41].

Since the scalene gap is not an empty space but is filled with connective and fatty tissue between the scalene muscle fascias and the prevertebral fascia, as well as around the spinal nerves, the spread of LA volumes injected in this area is unpredictable or uncontrollable. An LA distribution inside a “brachial plexus sheath”, as historically cited repeatedly, is more a wishful thought than an anatomically reproducible fact [42].

### 3.2. Interscalene Access

Ultrasound guidance is increasingly being used for ISB performance, whether through an in-plane (IP) or out-of-plane (OOP) technique. The OOP puncture technique, which is frequently used in practice, comes very close to the original landmark-based trajectory, according to Winnie [43]. In the age of ultrasound, clinicians should be aware that because of the round nature of the neck, an ultrasound beam targets medially, consequently resembling the pathway of Winnie if the canula is advanced perpendicularly to the beam. Therefore, the complications known from Winnie’s technique (subarachnoid, epidural, or vertebral artery injection) can also occur despite the use of sonography. This can be avoided if the needle is advanced in a slide-down technique from medial to lateral directions while shifting the probe along the course of the spinal nerves.

### 3.3. The Risks of Interscalene Catheters

**Figure 7:**
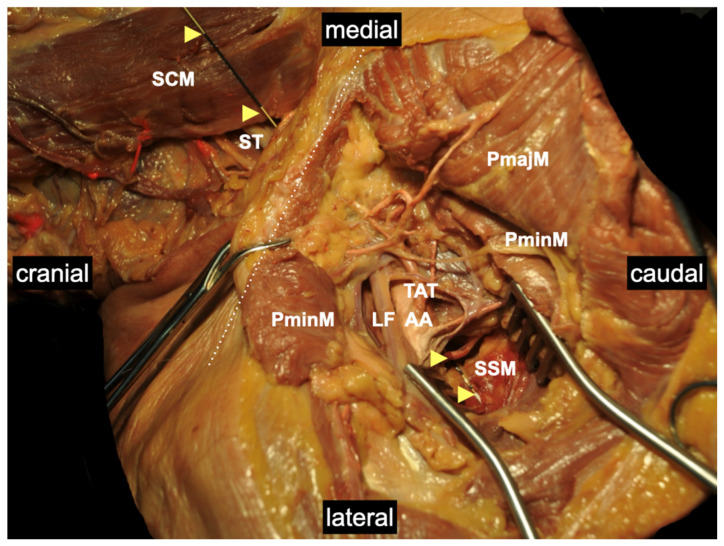
Anatomically plausible pathway of an interscalene-placed catheter directed toward the infraclavicular region demonstrated with a measuring pin. Yellow arrowheads mark the measuring pin that can be advanced from the interscalene to infraclavicular regions without significant resistance along the course of the brachial plexus in a preformed space. AA: axillary artery; LF: lateral fasciculus of brachial plexus; PmajM: pectoralis major muscle; PminM: pectoralis minor muscle; SCM: sternocleidomastoid muscle; SSM: subscapularis muscle; ST: superior trunk; TAT: thoracoacromial trunk. Details of the body donor’s embalmment, preparation, and dissection are provided in Appendix A.

If catheters are advanced, they can also deviate medially toward the neuroforamen or caudally in the direction of the scalenovertebral triangle and thus reach the pleura (Figure 4) [44]. This can explain inter-/intrapleural catheter malpositions if the catheter is being threaded too far or by a secondary catheter shift. On the other hand, if the catheter is advanced under the prevertebral fascia in a lateral direction, it can follow an anatomically preformed space with loose connective tissue in an infraclavicular/axillary direction (Figure 7) [39]. This corresponds to the landmark-based techniques described by Borgeat [45] and Meier [46]. Both techniques aim in a lateral direction and not toward the neuroforamina like Winnie’s original technique. They are therefore less prone to complications.

The connective tissue, the ventral rami, and vessels in the scalene gap are obstacles during catheter advancement, making it so that a catheter cannot always be reliably placed in the target position. Catheter advancement several centimeters beyond the canula tip is therefore frequently enforced in this area. If doing so, the definite course and final position of the catheter are speculative. A straight-line course within a predefined plexus sheath is anatomically impossible. Hence, a course consisting of moving the catheter in the direction of the neuroforamina is almost to be expected if the catheter has advanced too far [2,19].

### 3.4. Awake or Asleep Interscalene Blocks?

For decades, anesthetists have believed that performing blocks and inserting catheters are safer for awake patients. This is underlined by the case reports of Benumof and recommendations by anesthesia societies [18,47]. We have been performing ultrasound-guided blocks on thousands of anesthetized patients for decades. We have observed that patients more often agree to regional blocks if they are carried out under deep sedation or general anesthesia. For children, a puncture under deep sedation or anesthesia is generally accepted [48], whereas for adults, only awake punctures have been recommended in line with the relevant guidelines [47]. On closer inspection, the pediatric recommendations of the ASRA apply to “children” up to 18 years of age. In this context, it is inexplicable to anxious adult patients why different “safety standards” should apply to them compared to children, whose far smaller anatomical proportions and resulting shorter distances place even greater demands on the skills of a clinician [49]. The fact that touching nerves with the cannula does not necessarily trigger paresthesia has been repeatedly reported and can be regularly observed in routine sonography. Therefore, the absence of paresthesia cannot be taken as evidence of an extraneural and, thus, supposedly safe injection. If the basic rule “Without canula visualization: no needle advancement, no LA injection” is maintained, an increase in complications is not to be expected among asleep patients. According to the data available to date, it cannot be proven that there is a higher safety margin for awake patients [49].

In his excellent review, Marhofer pointed out myths and facts about regional blocks carried out under sedation or general anesthesia [49]. He concluded that with the use of ultrasound guidance in skilled hands, it is a reasonable option to carry out blocks in anesthetized or sedated patients. We fully support this statement, especially since interscalene catheter placements are often perceived to be unpleasant by awake patients.

### 3.5. Late Onset of Side Effects and Complications

It must be considered that side effects and complications can also occur late and at any time during continuous LA application. It is well known that, in principle, catheters can dislocate during the postoperative course, particularly during movement or physiotherapy [25,26,44,50]. If a reduction in effectiveness occurs over time during continuous catheter analgesia, the diagnosis is clinically simple. However, sonographic examinations have also shown that changes in catheter position occur in situ, even without a loss of efficacy [26]. As recommended by Gaus et al. [2], in order to detect such malpositions in a timely manner, continuous care must be ensured until the end of catheter therapy. In addition, patients must be informed about such unpredictable catheter dislocations and the possibility of the late occurrence of side effects or complications.

In a study involving 1505 outpatients, 27% of patients reported shortness of breath at home, while 13% and 7% reported hoarseness and dysphagia, respectively [51]. This clearly shows that side effects are almost inevitable, even in the late course, whether due to unnoticed catheter dislocation or simply the cumulatively applied LA volumes. In an anatomical study, a mean volume of only 4 mL was determined for the scalene gap [52]. This volume is higher than in the living, as connective tissue, nerves, and vessels are removed. In addition, the prevertebral fascia was removed, too. The fascia acts as the ventral boundary of the scalene gap and is therefore important for LA distribution. For example, a continuous rate of only 4 mL/h of LA results in 96 mL/day. Additional bolus function increases the volume even more. This amounts to a huge volume in the small scalene gap. The route the LA will take over time depends crucially on the anatomical pathways, the consecutive pressure conditions in the compartment, and the resorption rate at the application site. However, this highly individual resorption rate is ultimately unknown.

### 3.6. The Role of Ultrasound

**Figure 8:**
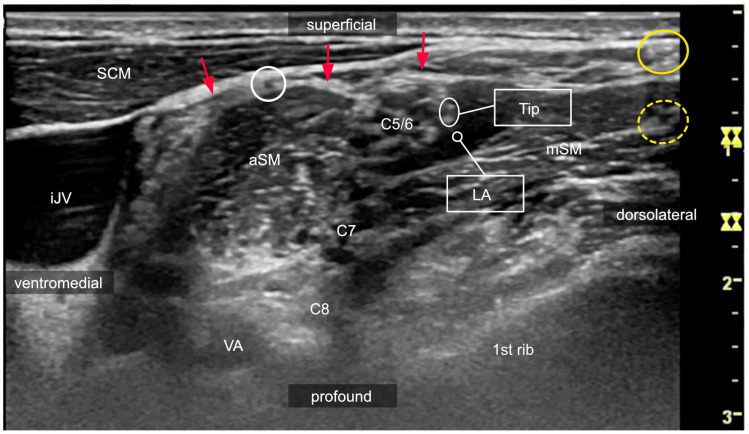
Ultrasound image captured at the level of the first rib, where spinal nerves C5 and C6 form the superior trunk, demonstrating the instant control of LA spread observation. The needle tip position can be controlled by the “double-dot sign”, which represents the orifice of the needle with the bevel up (white oval circle). In this patient, the out-of-plane approach was used to place a catheter in the direction of the plexus and sidestep the dorsal scapular nerve (yellow dotted circle), which could interfere with the in-plane approach from the posterior–lateral direction. LA injection can be stopped if one observes medial spreading toward the phrenic nerve (white circle). One branch of the supraclavicular nerve is marked by a yellow circle. If the supraclavicular nerves need to be blocked, the needle must be withdrawn and redirected because the prevertebral fascia (red arrows) represents a mechanical barrier for LA. aSM: anterior scalene muscle; mSM: middle scalene muscle; iJV: internal jugular vein; VA: vertebral artery; C7 and C8: ventral rami of the 7th and 8th spinal nerves, respectively. SCM: sternocleidomastoid muscle.

Modern ultrasound probes, such as those used for ISB, offer an axial and lateral resolution of approximately 0.4 mm and 0.7 mm, respectively, at a depth of 2 cm and a transmission frequency of 12–15 MHz. They allow extremely precise needle guidance and positioning. However, such high puncture precision can only be achieved by experienced operators using an excellent sonography technique. It is important to move the ultrasound probe in small steps in order to generate a sonogram that appears almost fixed. One way to train in the scanning of the lateral region of the neck is the so-called traceback technique [53]. Ultrasound helps to visualize LA spread, too. Physicians can stop the injection if LA spreads toward an undesired direction or proceed if the distribution is correct (Figure 8).

**Figure 9:**
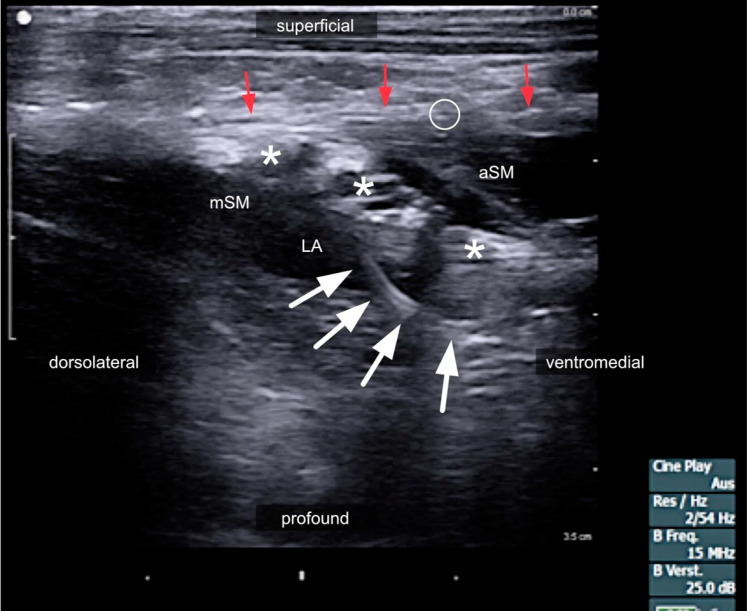
Sonogram of the left lateral neck region after the injection of 1 mL of local anesthetic via an interscalene catheter. White stars: brachial plexus; red arrows: prevertebral fascia; white arrowheads mark the catheter in situ; white circle: phrenic nerve; LA: local anesthetic; aSM: anterior scalene muscle; SCM sternocleidomastoid muscle; mSM: middle scalene muscle.

As discussed by Gaus et al. [2], the sonographic visualization of a catheter in situ is not easy, but it is certainly possible [26]. Catheters are difficult to visualize in situ in a single acoustic plane. Due to their anatomically determined, usually non-linear course, they cause significantly lower reflection than a straight cannula. The use of ultrasound-optimized catheters, which have recently become available (Figure 9), also facilitates catheter identification. However, if a catheter is advanced in an uncontrolled manner several centimeters beyond the visible cannula tip, identification of the catheter tip is almost impossible if the catheter tip has already reached the neuroforamen or the scalenovertebral triangle. In a study evaluating the dislocation rates of ISC among volunteers, Marhofer et al. advanced a catheter 3 cm beyond the needle tip and retracted it until a slow injection of saline confirmed an optimal spread pattern at the ventral rami C5–7, thus excluding intrathecal placement [25]. Such a procedure seems to be the most reasonable technique but is rarely addressed in the literature; thus, it is probably not performed by most practicians. Many anesthetists believe that catheter advancement several centimeters beyond the cannula tip prevents dislocations. In our own study using self-coiling catheters, catheters were advanced 2 cm beyond the tip until the coil was outside of the cannula. The correct position was confirmed sonographically [26]. The technique of using catheter depiction to sonographically confirm correct positioning was also used by others [54]. Despite an initially correct placement, it was demonstrated that catheters dislocated subcutaneously in the postoperative course without presenting externally visible signs. To give the catheter a certain stability in situ with a short puncture path to the target, subcutaneous tunneling can be performed instead of pushing the catheter beyond the target area in an uncontrolled manner [25]. A flat puncture angle with a lateral and caudal puncture direction corresponding to the anatomical course of the spinal nerves can also lead to a more stable catheter position in situ (Figure 9) [39]. However, clinical confirmation with larger case numbers is still lacking in this regard.

## 4. Limitations

The limitations of this review include the insufficient availability of data on prospective and blinded studies on interscalene catheters that were sonographically monitored postoperatively and over time. While the methodologies of individual studies occasionally provide more or less detailed descriptions of ultrasound-guided punctures, there is a lack of anatomical richness in the details. The anatomical foundation with which the complications and side effects of interscalene catheters can be explained was the motivation behind writing this article, aiming to encourage the consideration of such details in the future.

## 5. Summary

Interscalene analgesia is still considered the gold standard for the treatment of postoperative pain after shoulder surgery. This technique provides excellent analgesia but is associated with frequently occurring side effects and even life-threatening complications. To date, the evidence of alternative techniques for continuous postoperative analgesia is sparse. Profound anatomical knowledge is the key to understanding why side effects and complications of brachial plexus blocks at the interscalene level may occur. Currently, ultrasound is the best regional anesthesia strategy with which to face the complex demands of regional anesthesia of the lateral neck; identify individual variable anatomy; perform safe punctures; observe correct LA spread; detect (mis-)placed catheters; and therefore provide best practices to patients scheduled for shoulder surgery. Profound manual skills and knowledge of the relevant spaces and potential pathways of local anesthetics are the indispensable basis for safe and effective blocking techniques, thus avoiding travel between Scylla and Charybdis.

## Data Availability

Data is contained within the article, further inquiries can be directed to the corresponding author.

## Appendix A

Details of body donors and cadaveric specimens

All photos of cadaveric specimens came from bodies donated to science, specifically to the Department of Anatomy of the Medical University Graz, under the approval and strict rules of the Anatomical Donation Program of the University of Graz and according to the Styrian burial law. The bodies donated to science were analyzed and dissected between 2014 and 2018 and embalmed with Thiel’s method [55]. This embalming method creates flexible cadavers with almost lifelike conditions [56]. Cross or sagittal sections were made on embalmed cadavers that were frozen and then cut with a bandsaw. After cutting, the sections were thawed, documented via photography using a Nikon D 90 (Tokyo, Japan), and partially dissected to trace and identify several specific structures such as the prevertebral fascia, phrenic nerve, or ventral rami of the brachial plexus (Figure 1, Figure 2 and Figure 5). Dissection images were collected during investigations performed in the already-mentioned timeslot (Figure 6 and Figure 7). The anatomical knowledge expressed herein is based on the experience of Georg Feigl during his dissections conducted while working for his former employer, the Institute of Macroscopic and Clinical Anatomy, Medical University of Graz, Austria, and those conducted now at the Institute of Anatomy, University of Witten/Herdecke, Germany.

Scylla and Charybdis

The idiom “being between Scylla and Charybdis” is derived from Greek mythology and means to be in a quandary. Homer’s Odyssey described Scylla and Charybdis as two mythical sea monsters in the Strait of Messina. They were located opposite each other between the island of Sicily and the region of Calabria on the Italian mainland. Scylla was described as a six-headed sea monster (most likely a rock shoal) on the Calabrian side of the strait that would devour sailors who came too close. Charybdis was described as a whirlpool off the coast of Sicily that would devour entire ships. They were described as maritime hazards located close to each other, thus posing an inescapable threat to passing sailors. Avoiding Charybdis means passing too close to Scylla and vice versa.

The idiom “being between Scylla and Charybdis” describes the proverbial advice “to choose among two evils in favor of the lesser one”. According to Homer’s account, Odysseus was advised to pass by Scylla and lose only a few sailors rather than risk the loss of his entire ship in the whirlpool.

This mythical situation also assumed a proverbial use in which seeking to choose between equally dangerous extremes is seen as leading inevitably to disaster. The idiom “between Scylla and Charybdis” has come to mean being forced to choose between two similarly dangerous situations.
